# Atypical Type A Thymoma Identified in Slowly Growing Pulmonary Metastases from Type A Thymoma: A Case Report

**DOI:** 10.1159/cro/abzag008

**Published:** 2026-08-14

**Authors:** Hiroshi Shintani, Satoshi Wakama, Katsuji Tokuhara, Naoki Kataoka

**Affiliations:** Department of Surgery, Kishiwada Tokushukai Hospital, Osaka, Japan

**Keywords:** Atypical type A thymoma, Mediastinal neoplasms, Pulmonary metastasis

## Abstract

**Introduction:**

Type A thymoma is generally considered a low-risk thymoma subtype with an excellent prognosis and low rates of distant metastasis. Atypical type A thymoma is a recently established entity characterized by more aggressive biological behavior despite retaining the morphology of conventional type A thymoma. Patients with primary conventional type A thymoma who subsequently develop recurrence with atypical histological features are extremely rare.

**Case Presentation:**

A 79-year-old man was referred to our hospital due to gradual enlargement of bilateral pulmonary nodules. Seven years earlier, he had undergone complete resection of an anterior mediastinal tumor, which had been diagnosed as conventional type A thymoma. Retrospective review of preoperative computed tomography images revealed that small pulmonary nodules had already been present at the time of the initial surgery but had yet to be identified. The nodules enlarged slowly from 3 mm to 10 mm and from 3 mm to 7 mm over the 7-year period. Positron emission tomography showed no abnormal fluorodeoxyglucose uptake. After thoracoscopic wedge resection of the left lower lobe lesion, histopathological examination demonstrated pulmonary metastasis of type A thymoma with focal hypercellularity and increased mitotic activity, consistent with atypical type A thymoma. No necrosis was identified. The Ki-67 labeling indices of the primary and metastatic lesions were 7% and 20%, respectively.

**Conclusion:**

This case demonstrates that even apparently low-risk type A thymoma may exhibit indolent but metastatic behavior and may develop atypical histological features during a prolonged clinical course. Careful long-term surveillance and detailed pathological evaluation, including assessment of the Ki-67 labeling index, may be important in patients with type A thymoma.

## Introduction

Type A thymoma has generally been regarded as a low-risk subtype of thymoma with an excellent prognosis [1, 2]. Most patients present with localized disease, with distant metastasis being quite uncommon. Hence, long-term survival after complete surgical resection is generally expected.

Several studies have shown that a subset of type A thymomas exhibits more aggressive biological behavior despite retaining the characteristic morphology of conventional type A thymomas [3–6]. This entity, known as atypical type A thymoma, has recently been recognized as a distinct subtype in the 5th edition of the World Health Organization (WHO) classification of Thoracic Tumours published in 2021 [6]. Although atypical type A thymomas have been associated with a higher risk of recurrence and metastasis than have conventional type A thymomas, reports remain limited owing to its rarity.

Even more rare are cases in which the primary tumor is diagnosed as typical type A thymoma, but the metastatic lesion subsequently demonstrates atypical histological features. We herein report a case involving a patient diagnosed with typical type A thymoma who developed pulmonary metastatic lesions showing atypical type A thymoma after a prolonged clinical course. This case report was prepared in accordance with the CARE reporting guidelines, and the completed CARE checklist is available as online supplementary material (for all online suppl. material, see https://doi.org/10.1159/cro/abzag008).

## Case Presentation

A 79-year-old man was referred to our hospital due to gradual enlargement of bilateral pulmonary nodules on follow-up computed tomography (CT). Seven years earlier, the patient had undergone thoracoscopic resection of an anterior mediastinal tumor detected incidentally during routine CT via the right thoracic approach. The patient had been asymptomatic, with no symptoms suggesting myasthenia gravis and negative findings on anti-acetylcholine receptor antibody testing. At the time of the initial surgery, chest CT demonstrated a heterogeneously enhanced anterior mediastinal mass measuring 48 × 35 mm (Fig. 1a). Comparison with a CT scan obtained 4 years earlier showed that the lesion had enlarged from 41 × 30 mm, indicating slow tumor growth over time (Fig. 1b). Macroscopic examination revealed that the resected specimen was a well-circumscribed encapsulated tumor measuring 47 mm in diameter (Fig. 2a). Histologically, the tumor was divided by fibrous septa. The tumor cells were oval- to spindle-shaped with minimal nuclear atypia. The spindle cells showed fascicular and storiform growth patterns, and hemangiopericytoma-like architecture was partially observed. Microcystic and glandular structures were also present. No accompanying lymphocytic infiltration was identified. Although focal intracapsular extension was observed, no apparent invasion into the surrounding adipose tissue was noted. The tumor was diagnosed as type A thymoma without atypical features. The Ki-67 labeling index of the primary tumor was 7%. The tumor was completely resected with negative surgical margins (R0 resection). Although the disease was initially considered localized, retrospective review of the preoperative CT images revealed bilateral pulmonary metastases that had already been present at the time of surgery. Accordingly, the disease was retrospectively classified as Masaoka-Koga stage IVb and cT1aN0M1b (stage IVB) according to the eighth edition of the TNM classification.

**Fig. 1.**
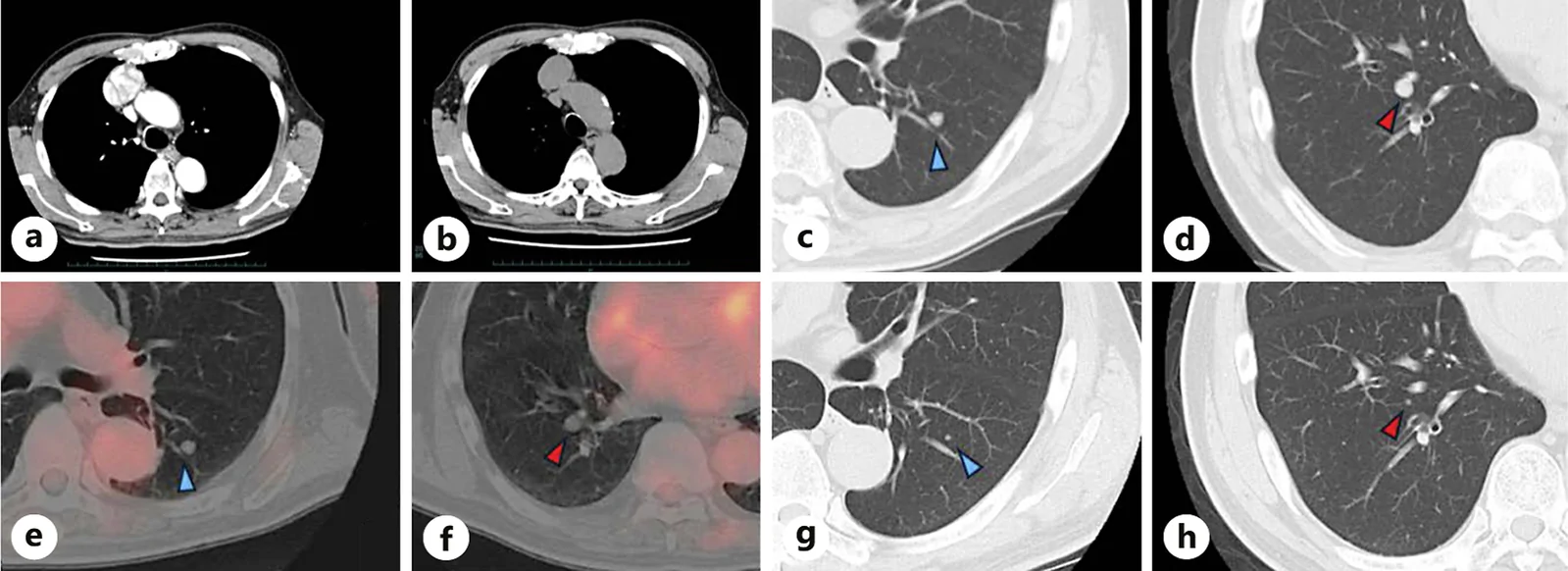
Radiological findings of the primary thymoma and pulmonary metastases. **a** Contrast-enhanced chest CT obtained 7 years before referral demonstrating a heterogeneously enhanced anterior mediastinal mass measuring 48 × 35 mm. **b** Chest CT obtained 4 years before the initial surgery showing an anterior mediastinal mass measuring 41 × 30 mm. **c** Chest CT (lung window setting) at referral demonstrating a 7-mm nodule in the left lower lobe (blue arrowhead). **d** Chest CT (lung window setting) at referral demonstrating a 10-mm nodule in the right lower lobe (red arrowhead). **e** Positron emission tomography at referral showing no abnormal fluorodeoxyglucose uptake in the left lower lobe nodule (blue arrowhead). **f** Positron emission tomography at referral showing no abnormal fluorodeoxyglucose uptake in the right lower lobe nodule (red arrowhead). **g** Retrospective review of chest CT obtained before the initial thymoma surgery demonstrating a 3-mm nodule in the left lower lobe (blue arrowhead). **h** Retrospective review of chest CT obtained before the initial thymoma surgery demonstrating a 3-mm nodule in the right lower lobe (red arrowhead).

**Fig. 2.**
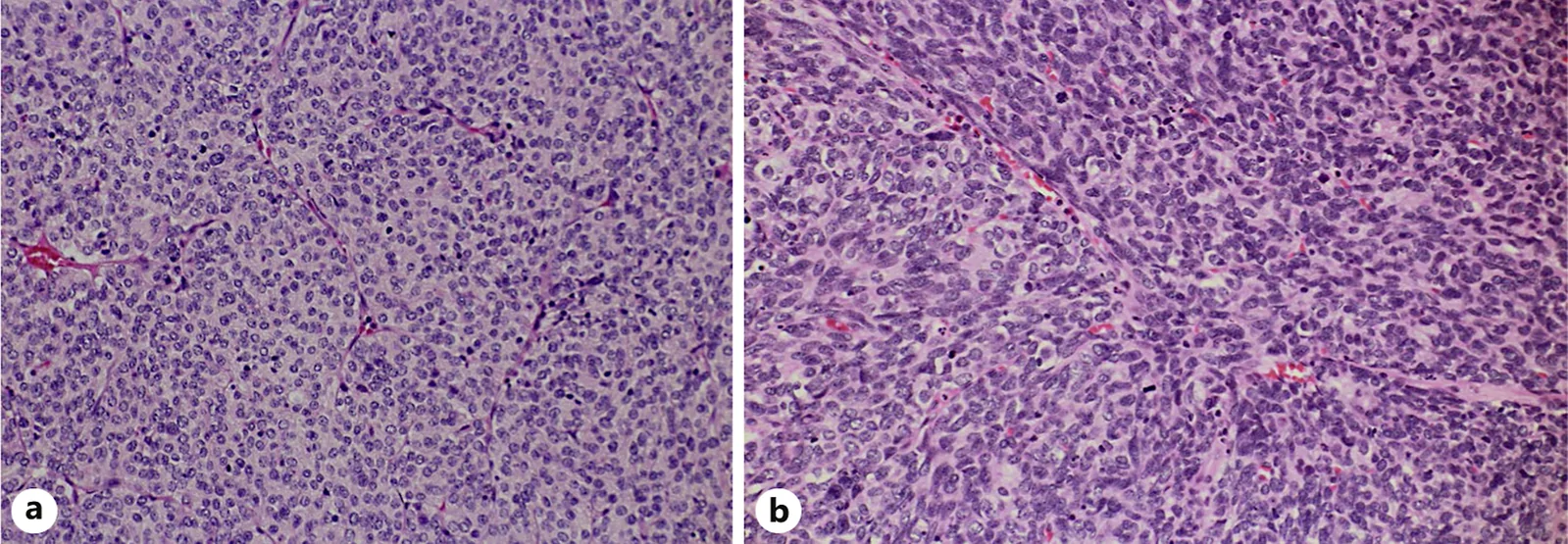
Histopathological findings of the primary and metastatic lesions. **a** Histopathological examination of the primary mediastinal tumor resected 7 years before referral. The tumor was composed of oval to spindle-shaped cells with minimal nuclear atypia, consistent with typical type A thymoma. **b** Histopathological examination of the pulmonary metastatic lesion resected at referral. Focal areas showed increased cellularity and increased mitotic activity, findings consistent with atypical type A thymoma.

Four years after the initial surgery, small pulmonary nodules were detected in both lower lobes on follow-up CT. Considering the extremely slow growth of the nodules, careful follow-up was continued. During follow-up, however, both bilateral lower lobe nodules gradually enlarged, promoting referral to our hospital. At the time of referral, the right and left lower lobe nodule had increased to 10 and 7 mm, respectively (Fig. 1c, d). Positron emission tomography showed no abnormal fluorodeoxyglucose uptake in either lesion (Fig. 1e, f). After referral to our hospital, a retrospective review of the preoperative CT images obtained before the initial surgery revealed that the same nodules had already been present at that time but had not yet been initially identified with both the right and left lower lobe nodules measuring 3 mm (Fig. 1g, h). Notably, no pulmonary nodules had been detected on CT performed 4 years before the initial surgery.

Given that the left lower lobe lesion located in segment 6 could be removed via limited wedge resection and that resection of the right lower lobe lesion would have required basal segmentectomy, thoracoscopic wedge resection of the left lower lobe was performed 1 month after referral. Macroscopic examination revealed that the resected pulmonary lesion was a relatively well-circumscribed tumor measuring 7 mm in diameter with central fibrosis (Fig. 2b). Histological examination showed that the lesion consisted of oval- to partially spindle-shaped tumor cells that were positive for p40 and showed sheet-like, fascicular, and interlacing proliferation. Focal microcystic formation was also observed. No lymphocytic infiltration was identified, consistent with pulmonary metastasis of type A thymoma. Furthermore, focal areas showed increased cellularity and mitotic activity, compatible with atypical type A thymoma. Notably, no necrosis was observed. Immunohistochemical analysis showed negativity for CD5 and CD117, with no findings suggesting thymic carcinoma. The Ki-67 labeling index of the metastatic lesion was 20%.

Despite the uneventful postoperative course, the patient experienced deterioration in activities of daily living during hospitalization, which prompted his transfer to another hospital for rehabilitation. Although resection of the remaining right lower lobe lesion was considered, careful follow-up was selected instead given the seemingly biologically indolent behavior of the residual tumor and the decline in the patient’s functional status. At 4 months after surgery, the residual right lower lobe nodule remained stable at 10 mm without obvious progression.

## Discussion

In the present case, the primary mediastinal tumor was initially diagnosed as typical type A thymoma and was surgically resected. However, retrospective review of the preoperative CT images later revealed that two small pulmonary nodules had already been present at the time of the initial surgery but had not yet been initially identified. Both nodules subsequently showed extremely slow growth over a 7-year period. After one of the metastatic lesions had eventually been resected, subsequent histopathological examination demonstrated atypical type A thymoma. To our knowledge, only a handful of reports are available on atypical type A thymoma. Even more rare are cases in which the primary lesion is diagnosed as typical type A thymoma, but the metastatic lesion demonstrates atypical features.

Atypical type A thymoma is a recently recognized subtype of type A thymoma that retains the characteristic morphology of conventional type A thymoma while exhibiting more aggressive biological behavior [3–6]. Histologically, it is characterized by hypercellularity, increased mitotic activity, and/or focal necrosis and has been associated with substantially higher recurrence rates than conventional type A thymoma [3–9]. Therefore, long-term postoperative surveillance has been recommended, including evaluation for extrathoracic recurrence [8].

In the present case, two pulmonary metastases had already been present at the time of the initial thymoma surgery. Although patients with type A thymoma usually present with early-stage disease, with approximately 90% having been diagnosed as stage I or II, those presenting with stage IVB disease are extremely rare and account for only 0.5% of cases [10]. Furthermore, fewer than 10 cases of pulmonary metastases from type A thymoma, including both typical and atypical subtypes, have been reported in the literature [11, 12].

Reports describing the growth rate of metastatic lesions are particularly limited. Kawagishi et al. [12] reported on a patient whose pulmonary metastasis enlarged from 13 to 18 mm over 14 months, corresponding to an annual growth rate of approximately 4.3 mm. Hirai et al. [8] described a case in which pulmonary metastases enlarged from 3 to 10 mm over 3.3 years, corresponding to an annual growth rate of approximately 2.1 mm. In the present case, two pulmonary metastases had already been present at the time of the initial thymoma surgery and subsequently enlarged very slowly over the following 7 years, with one lesion increasing from 3 to 10 mm and the other from 3 to 7 mm, corresponding to an annual growth rate of approximately 0.6–1.0 mm. Compared to the previous reports mentioned, the metastatic lesions in the present case demonstrated even slower growth. Studies have identified necrosis as an adverse prognostic factor in atypical type A thymoma [5, 8, 9]. Although atypical type A thymoma is characterized by hypercellularity, increased mitotic activity, and necrosis, the presence or absence of necrosis may be particularly important in determining biological behavior. In fact, Kawagishi et al. [12] reported histological necrosis in a rapidly enlarging metastatic lesion, whereas Hirai et al. [8] reported no necrosis in slowly growing metastatic lesions [8, 12]. These findings support the hypothesis that necrosis may be correlated with more aggressive clinical behavior. Similarly, the metastatic lesion in the present case showed no necrosis, with the pulmonary metastases demonstrating extremely indolent growth over a prolonged period. Our findings are therefore consistent with previous reports suggesting that the absence of necrosis may be associated with a more indolent clinical course.

Previous studies have suggested that atypical areas are often located adjacent to typical type A thymoma areas, potentially indicating that atypical type A thymoma may develop through progression from typical type A thymoma rather than arising de novo [13]. Similarly, the present case did present with atypical areas adjacent to typical areas within the metastatic lesion, consistent with previous reports. In the present case, the primary lesion was diagnosed as typical type A thymoma, whereas the metastatic lesion demonstrated atypical features. One possible explanation is that an extremely small atypical component had already existed within the primary lesion but had not yet been detected histologically. Another possibility is that part of the metastatic lesion acquired atypical features during the prolonged clinical course. Given that our patient was followed for 7 years after the detection of pulmonary metastases, the possibility that part of the typical lesion gradually progressed to atypical type A thymoma during follow-up cannot be excluded. However, the mechanism responsible for the gradual progression in the present case remains unclear. Nevertheless, this case suggests that some apparently low-risk type A thymomas may undergo biological progression and acquire atypical histological features during a prolonged clinical course. Clinicians should therefore consider the possibility of late recurrence and long-term indolent progression even in apparently low-risk type A thymoma. Furthermore, careful pathological evaluation may be important to identify focal atypical components within otherwise typical type A thymoma.

Ki-67, a well-established marker of cellular proliferation, has been widely used as an indicator of biological aggressiveness in various solid tumors. In fact, Hiroshima et al. [14] reported a correlation between the Ki-67 labeling index and tumor aggressiveness in thymic epithelial neoplasms. In general, type A thymomas have an exceedingly low Ki-67 labeling index, with the aforementioned study showing a mean Ki-67 labeling index of 1.2 ± 0.6% for type A thymoma [14]. However, no validated cut-off value of the Ki-67 labeling index has been established for distinguishing atypical type A thymoma from typical type A thymoma or for predicting aggressive clinical behavior. In the present case, the Ki-67 labeling index of the primary tumor was 7%, which was substantially higher than previously reported values for typical type A thymoma. Although Ki-67 is not included in the current diagnostic criteria for atypical type A thymoma, and the primary lesion could not be histologically classified as atypical type A thymoma, the elevated Ki-67 index may have reflected greater biological aggressiveness than expected for typical type A thymoma. As such, further studies are needed to clarify its prognostic significance. Nonetheless, careful long-term follow-up may be warranted in patients with type A thymoma showing an unusually elevated Ki-67 labeling index.

## Conclusion

We herein report a rare case involving a patient who presented with extremely slow-growing pulmonary metastases from typical type A thymoma in which the metastatic lesion demonstrated atypical type A thymoma after a prolonged clinical course. This case suggests that even apparently low-risk type A thymoma may exhibit indolent but metastatic behavior and develop atypical histological features over long-term follow-up. Furthermore, an elevated Ki-67 labeling index in the primary lesion may reflect greater biological aggressiveness than expected for conventional type A thymoma. Careful long-term surveillance and detailed pathological evaluation, including assessment of the Ki-67 labeling index, may therefore be imperative for patients with type A thymoma.

## Statement of Ethics

Ethical approval was not required for this case report, as per the policies of our Institution’s Ethics Committee. Ethical approval is not required for this study in accordance with local or national guidelines. Written informed consent for publication of the case details and accompanying images was obtained from the patient.

## Conflict of Interest Statement

The authors have no conflicts of interest to declare.

## Funding Sources

The researchers did not receive any specific grant from funding agencies in the public, commercial, or not-for-profit sectors.

## Author Contributions

Hiroshi Shintani contributed to the concept and drafting of the work, approved the final version to be published, and agreed to be accountable for the accuracy and integrity of the work. Satoshi Wakama, Katsuji Tokuhara, and Naoki Kataoka contributed to the work, assisted in the revision of the content, approved the final version to be published, and agreed to be accountable for the accuracy and integrity of the work.

## Data Availability

All data generated or analyzed during this study are included in this published article and its online supplementary material files. Further inquiries can be directed to the corresponding author.
